# iMetaOmics: Advancing human and environmental health through integrated meta‐omics

**DOI:** 10.1002/imo2.21

**Published:** 2024-08-08

**Authors:** Chun‐Lin Shi, Tong Chen, Canhui Lan, Ren‐You Gan, Jun Yu, Fangqing Zhao, Yong‐Xin Liu

**Affiliations:** ^1^ ANGENOVO Oslo Norway; ^2^ State Key Laboratory for Quality Ensurance and Sustainable Use of Dao‐di Herbs, National Resource Center for Chinese Materia Medica China Academy of Chinese Medical Sciences Beijing China; ^3^ School of Life Science and Technology Wuhan Polytechnic University Wuhan China; ^4^ R‐Institute Co. Ltd. Beijing China; ^5^ Department of Nutrition, Singapore Institute of Food and Biotechnology Innovation (SIFBI) Agency for Science, Technology and Research (A*STAR) Singapore Singapore; ^6^ Department of Food Science and Nutrition, Faculty of Science The Hong Kong Polytechnic University Hong Kong SAR China; ^7^ Institute of Digestive Disease and Department of Medicine and Therapeutics, State Key Laboratory of Digestive Disease, Li Ka Shing Institute of Health Sciences The Chinese University of Hong Kong Hong Kong SAR China; ^8^ Institute of Zoology Chinese Academy of Sciences Beijing China; ^9^ University of Chinese Academy of Sciences Beijing China; ^10^ Shenzhen Branch, Guangdong Laboratory of Lingnan Modern Agriculture, Genome Analysis Laboratory of the Ministry of Agriculture and Rural Affairs, Agricultural Genomics Institute at Shenzhen Chinese Academy of Agricultural Sciences Shenzhen Guangdong China

## Abstract

*iMetaOmics* is a quarterly international journal with the priority of publishing research work within the scope of One Health, featured with well‐designed omics. The journal also welcomes the work with systematic integration of extensive public datasets, new and meaningful perspectives, and the re‐analysis of high‐impact data yielding different/valuable conclusions.

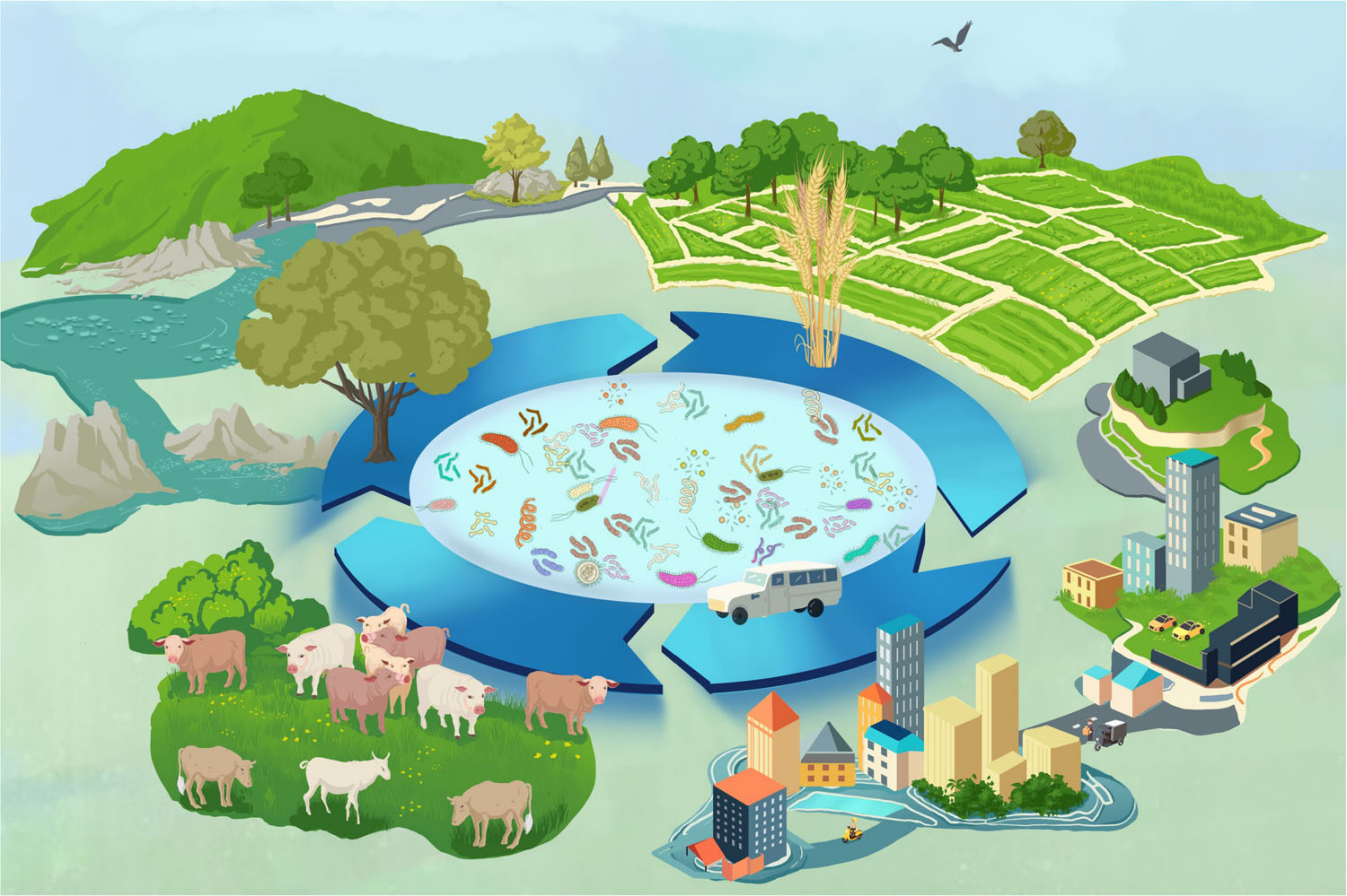

The microbiome plays critical roles in human [1], animal [2], and plant nutrition and health [3], food [4], environment [5], as well as other aspects of the human society (Figure 1). Metaomics, including metabarcoding, metagenomics, metatranscriptomics, metaproteomics, and metabolomics, provide a unique and powerful tools to better understand the taxonomy and functions of the microbiome, biology, and environment [6].

**Figure 1:**
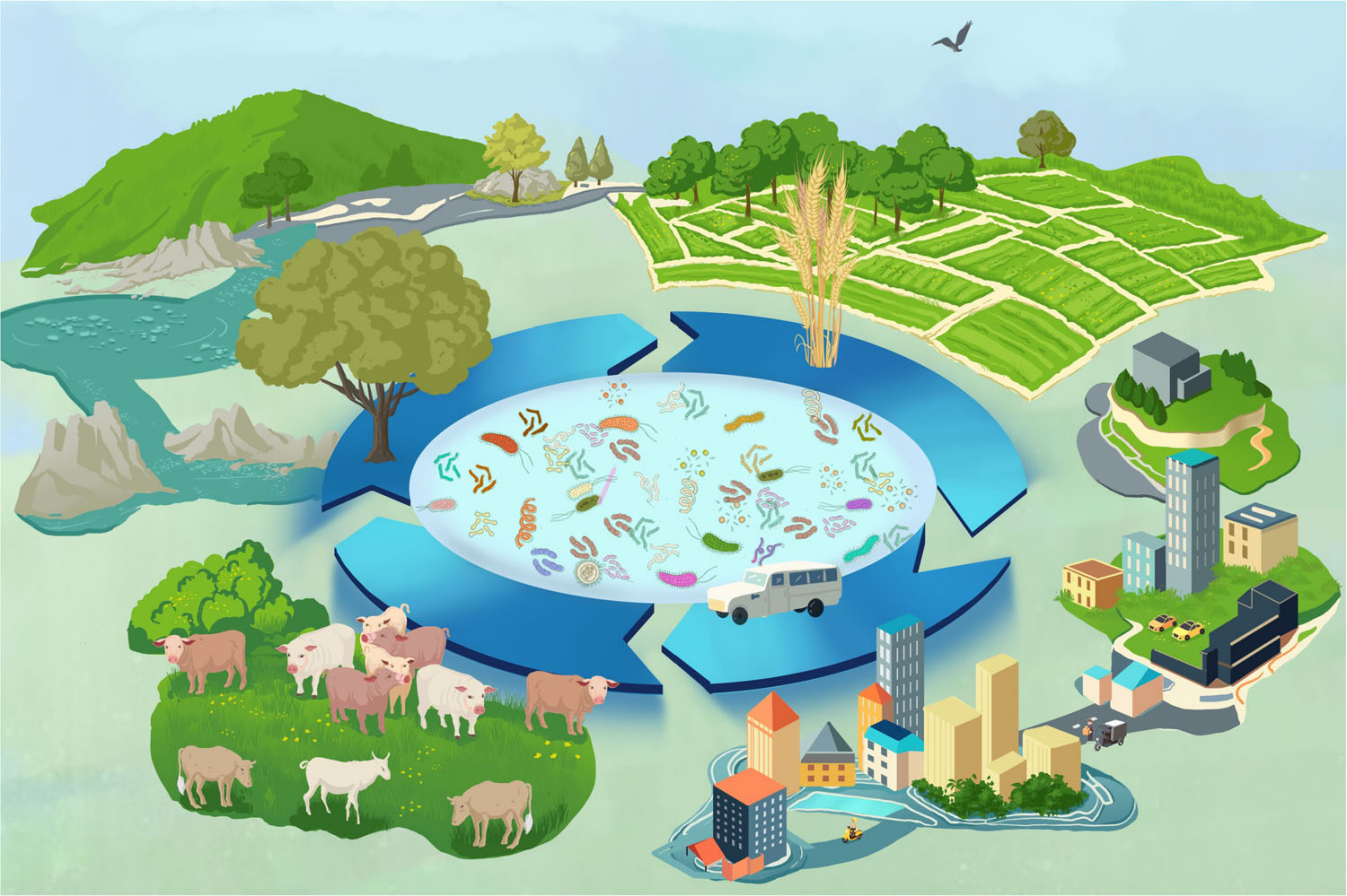
*iMetaOmics* as a powerful tool for One Health studies relative to the human, plant, animal, microbiome, and environment.

Aiming to fill in the gap of lacking top journals in the microbiome field in Asia, the iMeta Science Society and its thousands of Chinese scientist members found a young but high‐impact journal—*iMeta* (ISSN: 2770‐5986; eISSN: 2770‐596X). It has published more than 200 high‐quality papers since 2022, including original articles, reproducible methods and protocols, and systemic reviews to provide peers with updated and novel findings, easy‐to‐use analytic tools, and systematic knowledge [7]. These publications include but are not limited to gut microbiome pertaining to human diseases [8], microbiota in animals (e.g., chicken [9], pig [10], and panda [11]), pan‐cancer [12] and pan‐genome [13], soil and plant microbiome associated with plant diversity and defense [14, 15], as well as popular tools for metaomics analyses [16, 17, 18]. With the great efforts and contributions of our stakeholders, like the publisher, editorial board members, authors, and reviewers, *iMeta* has received its first impact factor of 23.7 in 2024, gradually accumulating broad and diverse audiences and readership, and especially attracting explosive growth of submissions in recent days. To cope with the significantly increased submissions, we have updated iMeta from a quarterly journal to a bimonthly journal. However, with the limited publication space and the priority of maintaining a high standard and high impact of *iMeta*, we still have to reject more than 90% of submissions, although many of them also did an excellent work.

Due to the success of *iMeta*, we are officially launching a sister journal of *iMeta*, *iMetaOmics* (eISSN: 2996‐9514, ISSN: 2996‐9506), to further publish high‐quality original articles [19, 20, 21, 22, 23], excellent reviews [24, 25, 26, 27, 28], and state‐of‐the‐art methodologies [29, 30] to satisfy the increasing submission demand for *iMeta*. Taking advantage of the same editorial team, rejected manuscripts from *iMeta* with valuable/meaningful findings will have the priority to be transferred to *iMetaOmics*, together with detailed comments from reviewers. In this context, the following review process in *iMetaOmics* will be significantly shortened to promote a rapid publishing. Before its official launch, 12 manuscripts have already been transferred from *iMeta* to *iMetaOmics* upon approval by authors in the first issue.

Currently, *iMetaOmics* is a quarterly international journal with the priority of publishing research work within the scope of One Health, featuring well‐designed omics. The journal also welcomes the work with the systematic integration of extensive public datasets, new and meaningful perspectives, and the reanalysis of high‐impact data yielding different/valuable conclusions. It is expected that our new journal *iMetaOmics* will establish its reputation soon, attract a wide range of interest from authors to readers, and significantly contribute to the research progress in Human and Environmental Health studies.

Following *iMeta*, here are a few tips for your success in *iMetaOmics*:

1. Authors' own data will have the priority, while the utilization and analysis of public data with robust experimental validation can also be considered.
2. Multiple analyses and evidence are required to support the same conclusion.
3. New analytic methods and strategies will be an advantage, but not indispensable.
4. All used data without reasonable concerns should be publicly/temporally accessible for reviewers and editors.
5. A video tutorial, including software download, installation, operation, and result displaying, is highly recommended for new bioinformatic tools.
6. Additionally, necessary ethics numbers, clear layouts, high‐quality figures, and significant findings are expected.

## AUTHOR CONTRIBUTIONS

**Chun‐Lin Shi**: Writing—original draft; writing—review and editing. **Tong Chen**: Writing—review and editing. **Canhui Lan**: Writing—review and editing. **Ren‐You Gan**: Writing—review and editing. **Jun Yu**: Writing—review and editing. **Fangqing Zhao**: Writing—review and editing. **Yong‐Xin Liu**: Writing—review and editing; conceptualization.

## CONFLICT OF INTEREST STATEMENT

Fangqing Zhao and Jun Yu hold the position of Editor‐in‐Chief for iMetaOmics. Yong‐Xin Liu, Tong Chen, Canhui Lan, Chun‐Lin Shi and Ren‐You Gan hold the position of Executive Editors for iMetaOmics.
